# The quality of life of HIV positive Transgender and homosexual population in Karachi, Pakistan

**DOI:** 10.12669/pjms.39.4.6445

**Published:** 2023

**Authors:** Aneela Hussain, Anum Rahim, Fozia Rajput

**Affiliations:** 1 Dr. Aneela Hussain, MBBS, FCPS (Internal medicine), FCPS (Infectious Diseases) Department of Infectious Diseases, Indus Hospital & Health Network, Karachi, Pakistan; 2 Dr. Anum Rahim, MBBS, MSc Epidemiology and Biostatistics Community Health Sciences, Aga Khan University Hospital, Karachi, Pakistan; 3 Ms. Fozia Rajput, BSc Psychology, MSc Sociology Department of Infectious Diseases, Indus Hospital & Health Network, Karachi, Pakistan

**Keywords:** Transgender, Human Immunodeficiency Virus, Homosexual, Karachi, Pakistan

## Abstract

**Objectives:**

To study the demographics and the quality of life of Transgenders and men who have sex with men (MSM) infected with Human Immunodeficiency Virus (HIV)

**Methods:**

A Cross sectional study conducted from 2019 to 2021 in the Indus Hospital Karachi. A 30 minutes interview was conducted among HIV positive homosexual participants.

**Results:**

Out of 100 patients enrolled 58% were transgender, 39% uneducated, 76% not supported financially by family, 20% reported hospital misconduct, 50% were commercial sex workers and 64% had anxiety. Our results also showed that increasing patient knowledge does not guarantee safe sex habits (p-value=0.82).

**Conclusion:**

We found declining psycho-social wellbeing in this population. Education and family support may help establish a good standard of living in them. More studies are needed in the Pakistani transgender population in order to understand their needs better.

## INTRODUCTION

Homosexuals are individuals who have sexual and romantic attraction to individuals from their own gender without having any birth gender identity crisis.^1^ Men who have sex with men (MSM) are a different population, they have been wrongly designated as transgender throughout various occasions in history.^2^ Transgender are those who have a combination of male or female genital anatomy at birth (intersexed ) or have ideas of identifying to a gender different to that assigned to them at birth, which can be a male to female (MTF) or a female to male (FTM) preference^3^,^4^ This preference may cause them to seek gender altering surgeries, hormone use, including injectable hormones with needle sharing.^5^ An MSM does not change their anatomical gender. In some European studies 0.5 to 0.9% people have identified to being a gender different to what they had been assigned at birth.^2^,^6^

Both of these populations have almost overlapping HIV vulnerabilities. However, the transgender population has an additional stress of over-coming stigma, discrimination and penalizing health care attitudes.^7^ This social level stigmatization leads to seeking sex work (protected versus unprotected) as a means of economic survival, increasing the risk of acquiring and spreading HIV.^7^ Apart from HIV, these populations suffer ill- treatment, depression, drug abuse which has a potential to affect their Anti-retroviral therapy (ART) outcomes and adherence .^7^

A San Franciscan study found 22.8% of MTF transgenders being afflicted with HIV in comparison to 24.3% MSM.^5^ This is a large number requiring attention. Another article, reviewing 25 studies emphasized that MTF transgender sex workers were at a greater risk for acquiring HIV 27.3% in comparison to cisgender sex workers (15.1% in men and 4.5% in women respectively ) which means that they need appropriate healthcare in order to control HIV spread.^8^

In Pakistan, a concentrated epidemic of HIV was declared in 2005, where transgenders were considered second to injectable drug users as a high risk group for HIV acquisition (National prevalence 6.4% out of 21% for HIV)^9^ Another study quotes transgenders as 17.5% of the entire HIV population in Pakistan and at an eight-fold higher risk for acquiring HIV.^10^ A study found a prevalence of 3.6% HIV in transgender sex workers (TSW) in Karachi compared to 27.6% in Larkana with only 50% compliance to condom use and a lack of knowledge regarding safe sex practices. This signifies their role as a bridging-population in HIV spread to not only MSM, but cisgender men and women. Sex work was an only mode of income due to social stigmatization and lack of other options. There is increased use of recreational drugs in this population during sexual intercourse which adds to HIV spread through the intravascular route.^9^ In another Pakistan-based study, 71% MTF transgender people were found to inject drugs with only 33.7% compliance to condoms.^10^

Expectedly, there were societal barriers to healthcare access. This points towards training of healthcare workers in empathy and also knowledge of this population’s special medical and ethical requirements.^9^ Adding to this, discriminatory attitude by health care providers was a notable finding in another Indian study.^3^

Coming to their mental health, a study conducted in India, a country which has similar social issues as Pakistan, found significant psychosocial illnesses in the MSM and MTF transgender communities (43% and 35.33% respectively) which led to high risk sexual behaviors.^11^

A Pakistan-based study on psychosocial issues in transgenders revealed rejection, physical and mental abuse by other communities and their biological families. This study associated all this with low self-esteem in 74% of the MFT transgender population. However, better psychological well-being, in comparison, was found in those living with their gurus (Transgender community head or leader) rather than with family, friends or alone on account of an accepting supportive environment.^3^ This study also highlighted lack of education which was associated with mental health issues.^3^ Our objective was to study the demographics and the quality of life of Transgenders and men who have sex with men (MSM) infected with Human Immunodeficiency Virus (HIV)

## METHODS

We conducted a cross sectional Interview based study at the HIV Clinic, The Indus Hospital, Karachi, Pakistan from January 2020 to December 2020 after the Ethics committee approval (Approval number: IRD_IRB_2019_06_005, Dated: 2/12/2019).

The transgender and MSM HIV positive patients, > 15 years of age, visiting the clinic were enrolled, randomly, after verbal and written consent. Those 15 years or less and not consenting to be a part of the study were excluded. The clinic counsellor conducted a face to face 30 minute interview with the participants in the clinic using a questionnaire. The questionnaire was in Urdu, the local language, comprising of 40 questions including mental health questions, financial status, family support and gender affirmation procedures. 100 patients were interviewed. Their answers were recorded and kept under lock and key.

## RESULTS

A hundred patients were enrolled for this study. Out of which 58% were transgender while the rest were MSMs. The baseline characteristics are shown in Table-I. The median (IQR) age of these individuals was 34.0 (36.0-40.0), 35% were married. Median (IQR) number of off springs were 3.0(1.0-4.0). Only 11% could attend the university while 39% were uneducated.

**Table-I:** Baseline characteristics.

Variable	N (%)	Variable	N (%)
Age		Monthly income	
N	100	None	15
Median (IQR)	34.0(36.0-40.0)	<5000	3
Gender		5000-10000	10
TG	54	11000-20000	37
MSM	46	21000-30000	27
Marital Status		31000-50000	8
Unmarried	58	Visit healthcare other than HIV clinic	86
Married	35	Medical attention given:	
Divorced/separated	7	Poor	3
Number of children		Worse than average	7
N	100	Inhumane	3
Median (IQR)	3.0(1.0-4.0)	empathetically	1
Education		Ease of getting hospital admission	
Uneducated	39	Yes	80
Primary Education	18	No	20
Secondary Education	32	Reason for no :	
University	11	Discrimination by staff	15
Hormonal injection for gender switch	31	Non-affording	3
Surgical Procedure for gender switch	1	Other patients become uncomfortable	2
HIV since		Having safe sex awareness	80
N	100	Age of awareness:	
Mean (SD)/Months	14.6(9.6)	<18	1(2.4)
ARV since		18-25	18(42.9)
N	100	25-35	18(42.9)
Mean (SD)/Months	14.2(9.6)	36-45	5(11.9)
Insights into sexuality (age in years)		Awareness given by	
<10	14	Partner	2(2.3)
10-20	62	Doctor	39(44.8)
20-30	20	Guru	1(1.1)
30-40	2	Internet/Media	1(1.1)
40-60	2	NGO	23(26.4)
Feelings of Shame/Guilt	23	Other	21(24.1)
Reasons:		Practicing safe sex	
Discrimination	7	Always	12
Feels abnormal	13	Sometimes	63
Abuse	3	Age of safe sex practice	
Family support	11	18-25	35(46.1)
Family is unaware	38	25-35	30(39.5)
Family reaction		36-45	9(11.8)
Anger	22	Above 45	2(2.6)
Sorrow	12	Number of partners	
Disbelief	1	None	27
Disowning	16	Single	13
Financial support from family	24	Multiple	60
Lodging sharing with:		IV drug use	2
Family	67	Needle sharing	2
Partner	3	Other STI diagnosed	36
Friends	7	STI treated	32
TG community	12	Recuurence of STI	1
Live alone	11	On ARV	94 (94.9)
Anxiety	36	Adherent to ARV	89
Suicide attempt	4	Dancing at gatherings as a profession	25
If yes how many times	4	Sex worker	50
Mean (SD)	2.5(1.3)		
Reason of suicide			
Family issues	4(100.0)		
Condom use during sex work	38(76.0)		
Content with life	83		

Sixty-nine individuals denied using hormonal injections for gender switch while only one person got a surgical procedure for it. Mean (SD) duration of having diagnosed with HIV was 14.6±9.6 months and the duration of usage of Anti-retroviral therapy was 14.2(9.6).

Around 62% had an insight to their sexuality between 10 to 20 years of age while 23% felt guilt-ridden on their sexuality, out of which 13 individuals felt abnormal. About 38% had their gender orientation from their families while those who told their families shared that their reaction included anger followed by being renounced as a family member, sorrow and disbelief.

Families of 76% held off financial support. 67% live with their families but without financial support. Out of the entire cohort, only 15 individuals had no monthly income and three had income less than PKR.5,000. The income of the rest ranges between PKR.5,000 to 50,000 per month.

When it came to healthcare conduct, 14 individuals felt that they are improperly treated, and the majority thought they were treated worse than cisgender patients. About 20% reported that they are not given hospital admissions on account of discrimination. About 4/5th of the study population had safe-sex awareness which they acquired either from the doctor (44.8%) or by some transgender volunteer organization (26.4%). Despite knowledge, only twelve individuals practiced safe sex and 60% had multiple sex partners.

Only two individuals were intravenous drug users and both shared syringes/drug ampules. Interestingly, 64% denied having STDs. Out of those, who were diagnosed 11.1% felt that they are not treated properly while 64% reported suffering from anxiety and four of them tried to commit suicide due to family related social issues. One-fourth of these participants danced at gatherings as a profession, while 50% were sex workers. Despite the stated concerns, 83% reported contentment with their lives.

Factors like emotional and financial family support, marital status, and the cohabitations were assessed for their association, if any, to the level of anxiety and adherence to ART. Interestingly, we found none of the above-mentioned factors statistically significant (Table-II).

**Table-II:** Assessment of factors of Anxiety and Adherence.

	Anxiety (Yes)	Anxiety (No)	P-value	Adherence to ARV (Yes)	Adherence to ARV (No)	P-value
Family support to the feelings			0.143^¥^			0.075^¥^
Yes	1(2.8)	10(15.6)		9(10.1)	2(18.2)	
No	20(55.6)	31(48.4)		43(48.3)	8(72.7)	
Family is unaware	15(41.7)	23(35.9)		37(41.6)	1(9.1)	
Marital Status			0.545^¥^			0.889^¥^
Unmarried	21(58.3)	37(57.8)		52(58.4)	6(54.5)	
Married	14(38.9)	21(32.8)		31(34.8)	4(36.4)	
Divorced/separated	1(2.8)	6(9.4)		6(6.7)	1(9.1)	
Financial support from family			0.198^¥^			0.289^¥^
Yes	6(16.7)	18(28.1)		23(25.8)	1(9.1)	
No	30(83.3)	46(71.9)		66(74.2)	10(90.9)	
Share the house with			0.254^¥^			0.918^¥^
Family	27(75.0)	50(62.5)		59(66.3)	8(72.7)	
Partner	2(5.6)	1(1.6)		3(3.4)	0	
Friends	3(8.3)	4(6.3)		7(7.9)	0	
TG community	2(5.6)	10(15.6)		10(11.2)	2(18.2)	
Live alone	2(5.6)	9(14.1)		10(11.2)	1(9.1)	

¥ Fisher Exact Test

Education status and its association with safe sex practices were also assessed as shown in Table-III. We found that increasing education does not guarantee safe sex practices among the patients (p-value=0.82).

**Table-III:** Education and safe sex practices.

	Do you practice safe sex (Yes)	Do you practice safe sex (No)	Do you practice safe sex (Sometimes)	P-value
Education				0.82^¥^
Uneducated	5(41.7)	12(48.0)	22(34.9)	
Primary Education	1(8.3)	4(16.0)	13(20.6)	
Secondary Education	4(33.3)	6(24.0)	22(34.9)	
University	2(16.7)	3(12.0)	6(9.5)	

¥ Fisher Exact Test.

## DISCUSSION

Our study population was on an average 34 years of age mostly unmarried and illiterate. In a Brazilian study, lower education level was associated with HIV acquisition and the reasons identified by them were discrimination and verbal /physical assaults at schools. This mandates education for sexual minorities if we are to halt spread of this infection.^12^ Another study also enumerated, less than a high school degree (which in our set up would mean middle –pass) to be an independent risk factor for HIV.^13^ Hence, emphasis on education is needed if we want to lower HIV in this key population and encourage them in finding other occupations. Around half of our study population were commercial sex workers which is a high-risk profession and is closely related to lack of education.

Most of our population have not used hormonal injections or gender switch surgeries which contrasts with many studies abroad. This is likely related to lack of such facilities in Pakistan and the financial position of the person seeking such a change. A study spanning 14 years, across the US found 10.9% gender switch surgeries (mostly white ethnicity) out of which 84% were genital surgeries.^14^ Another study, again US-based, though retrospective with a study population of 99, found 35% cases of gender re-affirming surgery, mostly in transgender men, with 25% cases of chest surgery, 13% genital surgery and 8% facial reconstruction.^15^ Furthermore, there has been an improved life quality in these individuals after gender re–assignment surgeries as studied by Wierckx et al.^16^ which is interesting though we may be able to look into this only when such surgeries are seeks and offered in our country .

Most transgenders coming to our clinic had an insight into safe sex and condom use and this knowledge was given by doctors and volunteer organizations, interestingly, not by their friends or gurus. Astonishingly, despite knowing safe sex, they still have high risk sexual behaviors and multiple sexual partners. Interestingly, around 37% of the population had more than 10 sex partners in a US-based study and another showed 19.7% lack of condom use. 13,17

A small number had guilt associated with them being” different” which is fascinating as there have been previous findings of guilt and shame in this population as well. This may be an important feature to look into during ART follow ups as it may also cause treatment adherence issues and effect their mental well-being.^18^,^19^ Interestingly, quite a few from our group said that they were happy with their lives with no regrets, though an Indian study has touched the topic and found a substantial number feeling remorse at their gender.^20^

Furthermore, only two people from our study group used injectable medications or shared syringes and drug voiles in contrast to a Dominican republic study which showed 26.1% drug use mostly linked to psychological trauma, sexual abuse, violence and attempted murder.^21^Another found approximately 78 amongst 392 MTF transgenders using intravenous drugs other than hormones, out of which 38 people shared syringes and 22 shared cookers or voiles.^13^Another interesting finding is that a large number denied having any other sexually transmitted disease (STD) which might be due to lack in identification of the illness timely as it’s a contradicting finding to other researches on the subject.^22^

Most in our population, did not have emotional or financial family support, followed by family unawareness of their orientation, when a disclosure to the family was made the commonest reaction was anger, followed by disowning them and sorrow, this is resonating with a study which shows lack of family support in 29.45% cases after finding out their sexual orientation.^23^ This can be taken as grounds for family counselling and support networking in our set up in the future. Despite this, a large number in our study population had average to good living conditions, as they were earning their own livelihood and mostly lived with their families, though they may not be supported emotionally or financially. Those who earned made an income of PKR11, 000 to 20,000 per month and had three meals a day which points towards a fair living status in a third world country.

Most admitted to being treated worse than cisgender individuals at the health care centers and were denied healthcare facilities on account of their gender identity which included refusal to give admission to inpatient care, as we have seen in prior researches.^3^,^9^This is another issue requiring work in our set up. We mostly saw adherence to ART in our population which contrasts with findings by Baguso et al.^24^

Most refused to have suffered from anxiety or depression in their lifetime, and the ones who did suffer had mild anxiety and depression. A minority attempted suicide with family related issues being the cause in our cohort though a study conducted in transgenders attending a conference showed 41% rates of suicide and the reason was transgender related violence.^25^ Another study quoted 32% suicide rates.^13^ Literature review shows staggering rates of depression, anxiety and suicide which is alarming and requires vigilance during counselling sessions for ART adherence related issues.^11^,^13^,^20^,^25^

### Limitations:

The sample size was small and mental-health was self-reported by the participant. Our study highlights the social, financial and psychological issues of this neglected population in Pakistan which adds to the limited local literature on the subject. Transgender population suffers from HIV at a higher level and an effort has be made on identifying associated factors.

### Suggestions:

Psychosocial wellbeing for this sexual minority depends on their basic right to education, equality, family support and social acceptance. We suggest family and group counselling on clinic visits at regular intervals. Attention must be given to providing equal education, job opportunities and medical facilities. This may impact the prevalence of HIV in this key population in Pakistan.

## CONCLUSION

This study found declining psycho-social wellbeing in this population. Education and family support may help establish a good standard of living for them. More studies are needed in the Pakistani transgender population in order to understand their needs better.

### Authors Contribution

**AH**: conception of the idea, designing the study, literature search, data compilation, write up and reviewing the article, responsible and accountable for the accuracy and integrity of the work

**AR**: data analysis, reviewing the article, write up

**FR**: data collection, compilation, data cleaning and management, reviewing the article.

## References

[1] American Psychological Association (2013). Sexual orientation, homosexuality and bisexuality. Answers to Your Questions:For a Better Understanding of Sexual Orientation and Homosexuality.

[2] Van Caenegem E, Wierckx K, Elaut E, Buysse A, Dewaele A, Van Nieuwerburgh F (2015). Prevalence of gender nonconformity in Flanders, Belgium. Arch Sex Behav.

[3] Akhtar M, Bilour N (2020). State of mental health among transgender individuals in Pakistan:Psychological resilience and self-esteem. Community Ment Health J.

[4] American Psychological Association (2015). Guidelines for psychological practice with transgender and gender nonconforming people. Am Psychol.

[5] Herbst JH, Jacobs ED, Finlayson TJ, McKleroy VS, Neumann MS, Crepaz N (2008). Estimating HIV prevalence and risk behaviors of transgender persons in the United States:a systematic review. AIDS Behav.

[6] Glen F, Hurrell K (2012). Measuring gender identity. Equality and Human Rights Commission Manchester.

[7] Poteat T, Scheim A, Xavier J, Reisner S, Baral S (2016). Global Epidemiology of HIV Infection and Related Syndemics Affecting Transgender People. J Acquir Immune Defic Syndr.

[8] Operario D, Soma T, Underhill K (2008). Sex work and HIV status among transgender women:systematic review and meta-analysis. J Acquir Immune Defic Syndr.

[9] Altaf A, Zahidie A, Agha A (2012). Comparing risk factors of HIV among hijra sex workers in Larkana and other cities of Pakistan:an analytical cross sectional study. BMC Public Health.

[10] Ming LC, Hadi MA, Khan TM (2016). Transgender health in India and Pakistan. Lancet.

[11] Chakrapani V, Newman PA, Shunmugam M, Logie CH, Samuel M (2017). Syndemics of depression, alcohol use, and victimisation, and their association with HIV-related sexual risk among men who have sex with men and transgender women in India. Glob. Public Health.

[12] Batista RL, Verduguez ED, Inacio M, Cunha FS, Marques MD, Gomes NLRA (2020). Impact of schooling in the HIV/AIDS prevalence among Brazilian transgender women. Arch. Endocrinol. Metab.

[13] Clements-Nolle K, Marx R, Guzman R, Katz M (2001). HIV prevalence, risk behaviors, health care use, and mental health status of transgender persons:implications for public health intervention. Am J Public Health.

[14] Canner JK, Harfouch O, Kodadek LM, Pelaez D, Coon D, Offodile AC (2018). Temporal trends in gender-affirming surgery among transgender patients in the United States. JAMA Surg.

[15] Kailas M, Lu HM, Rothman EF, Safer JD (2017). Prevalence and Types of Gender-Affirming Surgery Among A Sample of Transgender Endocrinology Patients Prior to State Expansion of Insurance Coverage. Endocr Pract.

[16] Wierckx K, Van Caenegem E, Elaut E, Dedecker D, Van de Peer F, Toye K (2011). Quality of life and sexual health after sex reassignment surgery in transsexual men. J Sex Med.

[17] Lee SW, Deiss RG, Segura ER, Clark JL, Lake JE, Konda KA (2015). A cross-sectional study of low HIV testing frequency and high-risk behaviour among men who have sex with men and transgender women in Lima, Peru. BMC Public Health.

[18] Schaefer LC, Wheeler CC (2004). Guilt in cross gender identity conditions:Presentations and treatment. J Gay Lesbian Psychother.

[19] Giordano S (2018). Understanding the emotion of shame in transgender individuals-some insight from Kafka. Life Sci. Soc. Policy.

[20] Ganju D, Saggurti N (2017). Stigma, violence and HIV vulnerability among transgender persons in sex work in Maharashtra, India. Cult Health Sex.

[21] Budhwani H, Hearld KR, Milner AN, McGlaughlin E, Charow R (2017). Transgender Women's Drug Use in the Dominican Republic. Transgender Health.

[22] Boehmer U (2002). Twenty years of public health research:Inclusion of lesbian, gay, bisexual, and transgender populations. Am J Public Health.

[23] Seibel BL, Silva DB, Fontanari A, Catelan RF, Bercht AM, Stucky JL (2018). The impact of the parental support on risk factors in the process of gender affirmation of transgender and gender diverse people. Front Psychol.

[24] Baguso GN, Gay CL, Lee KA (2016). Medication adherence among transgender women living with HIV. AIDS care.

[25] Maguen S, Shipherd JC (2010). Suicide risk among transgender individuals. Psychol Sex.

